# Therapeutics

**Published:** 1880-03

**Authors:** 


### THERAPEUTICS.

THE TOPICAL USES OF ERGOT.—(*The American Journal of Medical Sciences*, July, 1879.)

Dr. Dabney has used ergot in cases of pterygium with decided benefit, and has obtained excellent results from its application in pharyngitis. In the latter affection it is of especial use when the blood-vessels of the pharynx are enlarged and tortuous, and when the secretion is not very great. In those cases where the mucous membrane is thickened it acts much more slowly, and in acute cases it possesses no advantage over other remedies. In affections of the pharynx a combination of ergotin with tincture of iodine, as in the following formula, is especially efficacious: Ergotin, grs. xx.; tinct. iod. f. ʒj.; glycerine to make f. ℥j. m. To be applied to the pharynx freely twice a day with a camel's hair brush. In hypertrophy of the tonsils, which is so often an accompaniment of chronic pharyngitis, the same solution applied to the glands two or three times a day gives excellent results. In cervical metritis the following is serviceable: Ergotin (or solid extract of ergot) grs. xx.; extract of belladonna, grs. ij.; cocoa butter q.s. M. Make into six suppositories, and insert into the vagina every night after using the hot douche. In warm weather a solution of ergotin and extract of belladonna in glycerine and water may be used in place of the suppositories, as in the following formula: Ergotin, ʒss.; extract of belladonna, grs. vj.; water and glycerine āā. f. ℥iv. m. A pledget of cotton is to be saturated with this solution and inserted into the vagina at bed-time after the hot douche. The cotton should of course be removed in the morning.

THE PHYSIOLOGICAL AND THERAPEUTICAL EFFECTS OF SALICYLIC ACID AND ITS COMPOUNDS.—(*New York Med. Journ.*, July and August, 1879.)

In an interesting paper, Dr. Moore sums up the recent advances in our knowledge of the physiological and therapeutic effects of salicylic acid. *In rheumatism* (1) its most beneficial effects are manifested in the more acute cases. In subacute cases there is less, and in chronic cases no advantage from its use. (2) It should be given in doses of not less than twenty grains, every two hours to an adult. (3) Its use should not abruptly terminate on the subsidence of the pain and fever, but the interval between the doses should be more and more prolonged. (4) By its employment "rheumatic fever" may, in the majority of cases, be made a disease of hours, instead of months, as it was formerly. (5) By the brevity of the febrile condition, the chances of the occurrence of cardiac complications are immensely diminished. *In typhoid fever* it is found (1) that salicylic acid and its salts have a decidedly beneficial effect in reducing the temperature, and therefore in decreasing the mortality. (2) That its tendency is to shorten the duration of the disease by its antiseptic properties. (3) That the salicylate treatment is equal to any other antipyretic form of medication. (4) That there is every reason to suppose that the salicylates, combined with the cold water treatment, would excel all other methods. (5) That a large dose of one drachm of salicylate of soda acts better given at once than in divided doses. Such a dose is sufficient for twenty-four hours. *In diphtheria* salicylic acid is a valuable agent, since it not only acts as an antiseptic, but also favors the exfoliation of the membrane from the throat by increasing the secretions behind it, and thus tearing it off.

It may be used either as a gargle or internally, or in both ways. It may also be employed beneficially in the form of spray, using a solution of the acid in alcohol and water, three grains to the ounce of fluid. The internal use of the salicylate should also be insisted upon. *In diabetes mellitus* the salicylate of soda given daily in doses of one hundred and twenty grains determines a decided diminution in the sugar excreted. The diminution of the saccharine element is liable to considerable variation, according to the class of case, but it is more remarkable by the greatest restriction of carbohydrates in the diet. Diminution in the quantity of urine is parallel with the diminution of sugar. Salicylic acid and its compounds have also been used with more or less success in the following diseases: Small-pox and scarlatina; dilatation of the stomach, mycosis œsophagi; yellow and intermittent fevers; pertussis; rheumatic iritis; neuralgia; dysentery; in ear diseases; pyæmia, erysipelas, surgical fever, etc.; and as an anaphrodisiac, in addition to its use in surgical practice.

THE ACTION AND USES OF HYOSCYAMIN.—(*The Lancet*, September and October, 1879.)

Mr. Prideaux analyses the various papers which have appeared upon the action and uses of hyoscyamin, the active principle of hyoscyamus. This substance, which was first obtained in 1873, is most reliable when it has been obtained as an extractive from the leaves. In its effects hyoscyamin approaches very closely to atropia. The ingestion of a small dose is followed by a reduction of the pulse-rate, accompanied by increased strength and a diminution of the respiratory act. An almost immediate quickening of the pulse-rate, with coincident central excitement, then occurs, followed by motor paralysis, and by loss of power over the co-ordinating centers, as shown by affections of the speech and vision. An hypnotic stage of greater or less duration then follows. Accompanying these symptoms there is always great dilatation of the pupil, hypermetropia, and dryness of the throat and skin. The drug is excreted by the kidneys, and to some extent by the intestinal canal. Death ensues in animals after large doses, through failure of the respiratory center, the heart's action continuing for a few seconds after the cessation of respiration. After a poisonous dose of hyoscyamin, the symptoms observed are: a great acceleration of the heart's action, steady fall of temperature, and rapid diminution of the respiratory movements, dependent probably on paralysis of the vagus nerve. In man, hyoscyamin acts as an hypnotic, producing sleep, sometimes of considerable duration, particularly in conditions of the brain where there is great excitement, as in mania, delirium tremens, meningitis, and where ordinary hypnotics, such as opium, are inadmissible; and others, as chloral, have failed or exhausted their effect. In acute mania, with a greatly impaired condition of bodily strength, a small dose (one-sixteenth of a grain) will produce a most marked action, and this is also the case in the excitement of senile dementia; whilst in the excitement of chronic mania large and frequently repeated doses are required to produce any effect. In the use of the drug the bodily condition of the patient must always for this reason be borne in mind. In chronic mania with exacerbations, hyoscyamin is very useful, cutting short the exhibitions of temper and excitement of a violent and destructive character. These cases, as a rule, require only one dose, and that a large one, viz., half a grain, or even one grain, to effect any good. In conclusion, Mr. Prideaux sums up the results which he has obtained by the use of hyoscyamin in the following words:

1. In most cases of mania, or where there exists great excitement of any aggressive or destructive character, or rapidity of movement and speech, the use of the drug is the most effectual and rapid means of exercising that form of restraint which has been known as "chemical restraint."

2. That in cases of acute mania it will produce sleep and quietude when all other drugs have failed, and is one of the most rapid and reliable narcotics which we possess.

3. That in the treatment of the epileptic status in epileptic mania it diminishes the number, frequency, and severity of the attacks, especially if its administration be extended over some time.

4. That in delusional insanity, especially in the mania of suspicion, and in other forms of mania where the delusions are varying and changeable, it has a decided action in producing such an altered condition of the cerebral status that a condition which has been termed "physiological mania" results, and this so eclipses the former delusions and hallucinations that they are forgotten and the mind becomes clear; while, if the subjection to the influence of the drug be continued, it ultimately leads, under favorable circumstances, to a permanent condition of quiescence, and restoration to a healthy state of mind.

5. That in chronic dementia, associated with destructive tendencies, bad habits and sleeplessness, the condition of the patient much improves after a continued course of small doses of the drug.

The disadvantages that have occurred from its use, and which have to be guarded against, are: the dryness of the tongue and pharynx that occurs, especially after a prolonged administration. This has been thought to contra-indicate its use in cases of artificial feeding, but provided the tube be dipped into an oily liquid before passing, I have not found it any inconvenience. The attacks of vomiting that have occurred, in some cases after administration for some weeks, necessarily leads to discontinuance of the drug. Vomiting occasionally occurs after one dose, even a small one, and in two cases mentioned by Dr. Lawson hæmatemesis took place. Where rapid and sudden action of the drug is feared in feeble cases, it is better to administer it with the food.

THE POISONOUS PROPERTIES OF CARBOLIC ACID.—(*Le Progrès Médical*, October 4, 1879.)

M. Binnendik read a paper before the Pharmacological section of the International Medical Congress recently held at Amsterdam, in which he discussed the poisonous properties of carbolic acid. The conclusions arrived at are: (1.) That pure carbolic acid is a poisonous substance which acts directly upon the cerebro-spinal nervous system, first stimulating and finally paralysing it. It does not appear to exert any influence upon the blood. Hæmoglobin however may often be detected in the urine for a short time after intoxication has been produced. (2.) The absorption and elimination of carbolic acid occur rapidly. The poisonous symptoms are developed very soon after the introduction of the acid, and they disappear before the whole quantity has been eliminated. Under favorable conditions, as after subcutaneous injection, or administration upon an empty stomach, the elimination is complete in twelve or sixteen hours. (3.) Carbolic acid undergoes a change in the organism. It forms complex chemical bodies, such as compound sulphuric ethers of phenol, hydroquinon, and the pyrocatechin of Baumann, which are of a less poisonous nature than the pure carbolic acid. A portion is also in all probability oxidized. (4.) Glycerine added to aqueous solutions of carbolic acid diminishes the poisonous effects in rabbits. It has not yet been determined, however, whether the glycerine acts by retarding the rate of absorption, or whether its modifying action is due to other circumstances.

HYPODERMIC USE OF COLCHICIN IN RHEUMATISM.—(*Berlin klin. Wochensch*, No. 26, 1879; *The New York Medical Journal*, June, 1879.)

The solubility of colchicin in water renders it very applicable for hypodermic use. Dr. Badia, a Spanish physician, has communicated a number of successful results after its employment in this way in chronic rheumatism, and these have induced Dr. Heyfelder, of St. Petersburg, to try it in a series of cases. The results were, on the whole, surprisingly gratifying. The remedy was tried in rheumatic joint affections, neuralgias of the same nature, and especially ischiagra. Two milligrams were injected, in one gram of water, and the action was often remarkable, the severe pain becoming much lessened, and the movability of the affected articulation being rendered much freer. In a case of chronic articular rheumatism of the lower extremity which had existed for years, a surprising result was obtained by daily injections for ten days. There was freedom from pain accompanied by greatly increased mobility; and a relapse having occurred, a similar result was obtained by a single injection. In rheumatic ischiagra, the remedy had generally a very pronounced effect in relieving pain. The first result of the injection is a severe burning pain, which rarely lasts more than half an hour. In about one-third of the cases there was a local inflammatory reaction of varying degree at the place of injection. In very few cases was there any considerable swelling or tenderness. Increased diuresis and strangury were noticed in a few cases. In persons with very sensitive skins it is advisable to use the remedy with great caution, and to diminish the dose. Where local inflammatory phenomena are present, the remedies should be discontinued, or applied at some distance.

TREATMENT OF DIPHTHERIA WITH BENZOATE OF SODA.—(*Berliner klin. Wochenschr.*, No. 7, 1879. *Cbl. f. med. Wiss.* Aug. 9, 1879.)

Dr. Letzerich has employed benzoate of soda in severe cases of diphtheria, as is recommended by Prof. Klebs. The dose administered to children under twelve months was a teaspoonful hourly of a five per cent. solution, for children of one to three years the same quantity of a seven-eighths per cent. solution, whilst to still older children, eight, ten and fifteen grams were given, adults receiving as much as fifteen-twenty-fifths grams a day. In no case did any untoward symptoms present themselves. The diphtheritic membrane was powdered with sodium benzoate, the local application being made every three hours in severe cases, and two or three times a day in less urgent ones. In every case the febrile symptoms were checked in from twenty-four to thirty-six hours, the temperature returning nearly to the normal. Dr. Letzerich recommends the employment of this remedy in catarrh of the stomach and intestines, especially in unweaned infants, in whose cases he has employed it with marked effects. It is also beneficial in mycotic catarrh of the bladder.

PECULIAR EFFECTS PRODUCED BY THE ADMINISTRATION OF HYDROCHLORATE OF MORPHIA.—(*British Medical Journal*, Oct. 18, 1879.)

Mr. Courtenay publishes the following case, which presents two interesting features:—1. The development of peculiar symptoms from the administration of morphia; 2. The marked physiological antagonism of belladonna to morphia, proved by the power of the former drug to speedily relieve the ill effects of the administration of the latter. A male coolie, under treatment for lumbago in the Lucea Parochial Hospital, Jamaica, was ordered a hypodermic injection of one-sixth of a grain of morphia, the injection to be repeated every night. This treatment was continued for three nights, and on the morning following the third use of the injection the patient complained of feeling unwell at breakfast, and retired to bed at 9:10 a. m., when he was siezed with convulsions of a tetanic nature. His symptoms were the following: He was quite insensible; his eyes were widely opened and staring, the pupils slightly contracted; teeth clenched, face flushed, nostrils dilated, and breathing hurried. There was spasmodic contraction of the muscles of the upper extremity; the temperature was increased, the heart's action regular, though weak. This attack lasted about ten minutes, when consciousness returned, but, on attempting to speak, he was siezed again, and the convulsions returned at gradually-increasing intervals, until they finally left him at twelve noon. The treatment immediately adopted in this case was the administration of doses of ten drops of the tincture of belladonna every hour, with coffee and brandy, and the application of the extract of belladonna down the course of the spine. This treatment was continued for a few hours, and all urgent symptoms having yielded to it, the belladonna was given in diminished doses of five drops every four hours, and continued for the two following days. At the end of that time, no recurrence of the convulsions having taken place, the ordinary treatment for lumbago was recommenced, of course with the omission of the morphia injection.

ERGOT IN THE TREATMENT OF FIBROUS TUMORS OF THE UTERUS.—(*Arch. génér. de méd.*, Nov., 1879.

A critical review on the important and pressing question as to the treatment of fibroid tumors of the uterus, recently appeared in *The Medical Times and Gazette.* In this paper Dr. Herman states that the method of administration of ergot is a point of practical importance. Hildebrandt recommends hypodermic injections of ergotin into the abdominal parietes in the neighborhood of the tumor. He believes that the drug thus administered acts more energetically than when given by the mouth, and he cites in proof several cases where the administration by the mouth failed to afford relief, whilst the subcutaneous injections were effectual. The experiments of Peton go far to prove that ergotin administered hypodermically exerts a definite local action, which is powerful and rapid in regard to the vessels in the immediate neighborhood of the injection, but is slow and feeble in the arteries of distant parts. This method has, however, the disadvantage of being painful, and it is difficult to exert sufficient control over its administration in the case of patients who are not seen every day. The effects of the two methods only appear to differ in intensity, and Dr. Herman therefore contents himself with giving the drug by the mouth. The inconveniences attending the use of ergot depend either upon a faulty preparation or upon idiosyncracy. The choice of the preparation of ergotin or sclerotinic acid appears to be immaterial, provided that the drug is fresh and is prepared in small quantities at a time.

BODENHAMER ON RECTAL MEDICATION.—(Pamphlet. New York: W. Wood & Co.—Abstract in *London Medical Record*, August 15, 1879.)

This essay is intended to draw more attention to the important subject of the administration of food and medicines by the rectum. The method is of great antiquity, and was recommended by Hippocrates, Galen and Celsus; but it has made little progress towards perfection, and the administration of enemata of any kind is often mischievously bungled by nurses, students, and even medical men. It is apparently forgotten that foreign substances introduced into the bowel are very apt to excite expulsive action, and therefore that extreme caution should be observed. The author recommends a screw syringe, which has the advantage that the enema cannot be suddenly shot into the rectum by a careless hand. Whatever instrument is used the fluid should be gently passed in, and so slowly that the bowel may have time to accommodate itself to the presence of the enema. After the administration of a medicated or nourishing enema, should there be a strong or irresistible desire to pass it, as is the case sometimes, when there exists an exquisitely irritable state of the organ, a sponge or fold of cloth dipped in hot water, and firmly pressed against the anus for a while, will generally appease the desire, and enable the patient to retain the enema. The author collects the opinions of some eminent men, and in the end lays down certain conclusions based upon the evidence quoted. He holds that a fuller and more rapid effect can be obtained in this way than by giving drugs by the mouth, and he points out that of narcotics the rectum only requires about one-third of the quantity necessary by the mouth. The book is an interesting contribution to practical medicine.

TREATMENT OF ASTHMA BY POTASSIUM IODIDE AND IODIDE OF ETHYL.—(*Le Progrés Médical*, Sept. 6, 1879.)

Dr. Gougeon has observed the beneficial results which occurred in certain cases of dyspnœa, after the use of potassium iodide, either alone or combined with iodide of ethyl. He has, therefore, in his inaugural thesis analyzed the works of M. Sée upon the subject. The monograph by Dr. Gougeon is a faithful and orderly *résumé* of the facts which have been observed. Every one knows how difficult it is to cure or even to benefit the majority of patients who suffer from asthmatic dyspnœa, whether the asthma be chronic or symptomatic. Many medicines have been proposed, but they have failed in their purpose, and have consequently fallen out of use. Prof. Sée, after a great number of investigations, has at last been led to adopt heroic treatment in nearly every case. He administers iodide of potassium at the outset of the disease in a sufficient dose, i. e., at least one and one-half grams, increasing it progressively to three to four grams, and only limiting the use of the drug on the appearance of symptoms of iodism. It is found that in this way alone is the action of the remedy real and effectual. In the historical part of his work, M. Gougeon shows, that before M. Sée turned his attention to the drug, it had been employed without any definite rules; and that it was the active principle in several secret remedies for asthma.

SULPHO-METHYLATE OF SODA: A NEW PURGATIVE.—(*La Praticien*, January 27, 1879.)

M. Rabuteau has investigated a new purgative, analogous to sulpho-vinate of soda. It is obtained by treating methyl alcohol with sulphuric acid, when methyl sulphuric acid and water are obtained. This product is neutralized with carbonate of barium, when the excess of sulphuric acid is thrown down in the insoluble form of barium sulphate, and there remains in solution sulpho-methylate of baryta, a substance which is very soluble, and which can be crystallized. It is then treated afresh with sulphuric acid, which liberates pure sulpho-methylic acid, which yields sulpho-methylate of soda when neutralized with soda. The salt is white and very soluble, crystallizing with difficulty, of a feeble taste comparable to that possessed by sulpho-vinate of soda, with a sweet after-taste. Unfortunately it decomposes very rapidly into sulphate of soda and methylic products of a slightly garlic-like odor. Ten grams of the salt in 25 c.c. of water injected into the veins of a dog produced constipation. Acting upon the observation that the substance, when introduced into the blood, gave rise to constipation, M. Rabateau believed that it should act as a dialytic purgative when given by the mouth. He therefore administered it to two patients; in one case a woman took 15 grams in two doses, when it produced three stools, of which two were copious; in the second, a man took 18 grams, resulting in two stools without colic. The taste of the purgative is hardly perceptible, but it is difficult to preserve it.

PLEURITIC EFFUSION TREATED WITH JABORANDI.—(*The Dublin Journal of Medical Science*, Nov., 1879.

Dr. Hunt has employed jaborandi in three cases of pleuritic effusion. In the first and second of these the remedy was only prescribed after the failure of other means of getting rid of the fluid which remained in the pleura following aspiration; and there could be very little doubt that absorption was materially hastened by its use. In the third case jaborandi was the sole drug that was tried, and therefore the results cannot be so conclusive. All the patients bore the jaborandi well: one, indeed, increasing in weight whilst he was sweating profusely under its influence. In no case did it lower the general health to any appreciable extent, and with the exception of the diaphoresis and salivation, there were no inconveniences attending its administration even in the largest doses (ʒjss secundis horis). In none of the cases was any beneficial result obtained until profuse diaphoresis had been excited.

